# Clinical outcomes of acute myocardial infarction arising from non-lipid-rich plaque determined by NIRS-IVUS

**DOI:** 10.1038/s41598-023-38578-9

**Published:** 2023-07-17

**Authors:** Kosei Terada, Noriyuki Wakana, Takashi Kubo, Yasushi Ino, Amir Kh. M. Khalifa, Suwako Fujita, Masahiro Takahata, Yasutsugu Shiono, Ryan D. Madder, Takeyoshi Kameyama

**Affiliations:** 1 Department of Cardiovascular Medicine, Wakayama Medical University, Wakayama, Japan; 2 Department of Cardiovascular Medicine, Kyoto Prefectural University of Medicine, Kyoto, Japan; 3 Division of Cardiology, Tokyo Medical University Hachioji Medical Center, 1163 Tate-machi, Hachioji, Tokyo, 193-0998 Japan; 4 Department of Cardiovascular Medicine, Shingu Municipal Hospital, Shingu, Japan; 5 Department of Cardiovascular Medicine, Assiut University Hospitals, Assiut, Egypt; 6 Frederik Meijer Heart and Vascular Institute, Spectrum Health, Grand Rapids, MI USA; 7 Department of Cardiovascular Medicine, Tohoku Medical and Pharmaceutical University, Sendai, Japan

**Keywords:** Cardiology, Interventional cardiology

## Abstract

Acute myocardial infarction (AMI) can rarely arise from non-lipid-rich coronary plaques. This study sought to compare the clinical outcomes after percutaneous coronary intervention (PCI) between AMI showing maximum lipid-core burden index in 4 mm (maxLCBI_4mm_) < 400 and ≥ 400 in the infarct-related lesions assessed by near-infrared spectroscopy-intravascular ultrasound (NIRS-IVUS). We investigated 426 AMI patients who underwent NIRS-IVUS in the infarct-related lesions before PCI. Major adverse cardiovascular events (MACE) were defined as the composite of cardiac death, non-fatal MI, clinically driven target lesion revascularization (TLR), clinically driven non-TLR, and congestive heart failure requiring hospitalization. 107 (25%) patients had infarct-related lesions of maxLCBI_4mm_ < 400, and 319 (75%) patients had those of maxLCBI_4mm_ ≥ 400. The maxLCBI_4mm_ < 400 group had a younger median age at onset (68 years [IQR: 57–78 years] vs. 73 years [IQR: 64–80 years], *P* = 0.007), less frequent multivessel disease (39% vs. 51%, *P* = 0.029), less frequent TIMI flow grade 0 or 1 before PCI (62% vs. 75%, *P* = 0.007), and less frequent no-reflow immediately after PCI (5% vs. 11%, *P* = 0.039). During a median follow-up period of 31 months [IQR: 19–48 months], the frequency of MACE was significantly lower in the maxLCBI_4mm_ < 400 group compared with the maxLCBI_4mm_ ≥ 400 group (4.7% vs. 17.2%, *P* = 0.001). MaxLCBI_4mm_ < 400 was an independent predictor of MACE-free survival at multivariable analysis (hazard ratio: 0.36 [confidence interval: 0.13–0.98], *P* = 0.046). MaxLCBI_4mm_ < 400 measured by NIRS in the infract-related lesions before PCI was associated with better long-term clinical outcomes in AMI patients.

## Introduction

Most acute myocardial infarctions (AMI) are caused by thrombotic occlusion of coronary atherosclerotic lesions. Atherosclerotic lesions have lipid deposits in the core region of the plaque. Previous autopsy studies have demonstrated that 50–70% of coronary thrombosis resulted from the rupture of a plaque with a large lipid-core^1^. However, 20–40% of coronary thrombosis resulted from non-ruptured plaques, generally with small or no evidence of a lipid-core^1^. Compared with AMI arising from the lipid-rich plaque, AMI arising from the non-lipid-rich plaque (defined as a lipid-free or small lipid-containing plaque) may have different clinical features and prognosis.

Developments of intravascular imaging have enabled evaluation of infarct-related coronary lesions of AMI in the clinical setting. However, intracoronary thrombi often obscure the lesion, preventing precise determination of the underlying mechanisms of AMI. Recently, near-infrared spectroscopy (NIRS) combined with intravascular ultrasound (IVUS) has emerged as an imaging technique that can accurately identify lipids in the coronary artery wall even in the presence of thrombi. Several NIRS studies have demonstrated that infarct-related lesions generally exhibit high lipid composition and rarely low lipid composition^2,3^. In NIRS, maximum lipid-core burden index in 4 mm (maxLCBI_4mm_) of 400 is used to differentiate between lipid-rich plaque and non-lipid-rich plaque^4^. The present study sought to compare the clinical outcomes after percutaneous coronary intervention (PCI) between AMI with maxLCBI_4mm_ < 400 and ≥ 400 in the infarct-related lesions assessed by NIRS.

## Methods

### Study population

**Figure 1:**
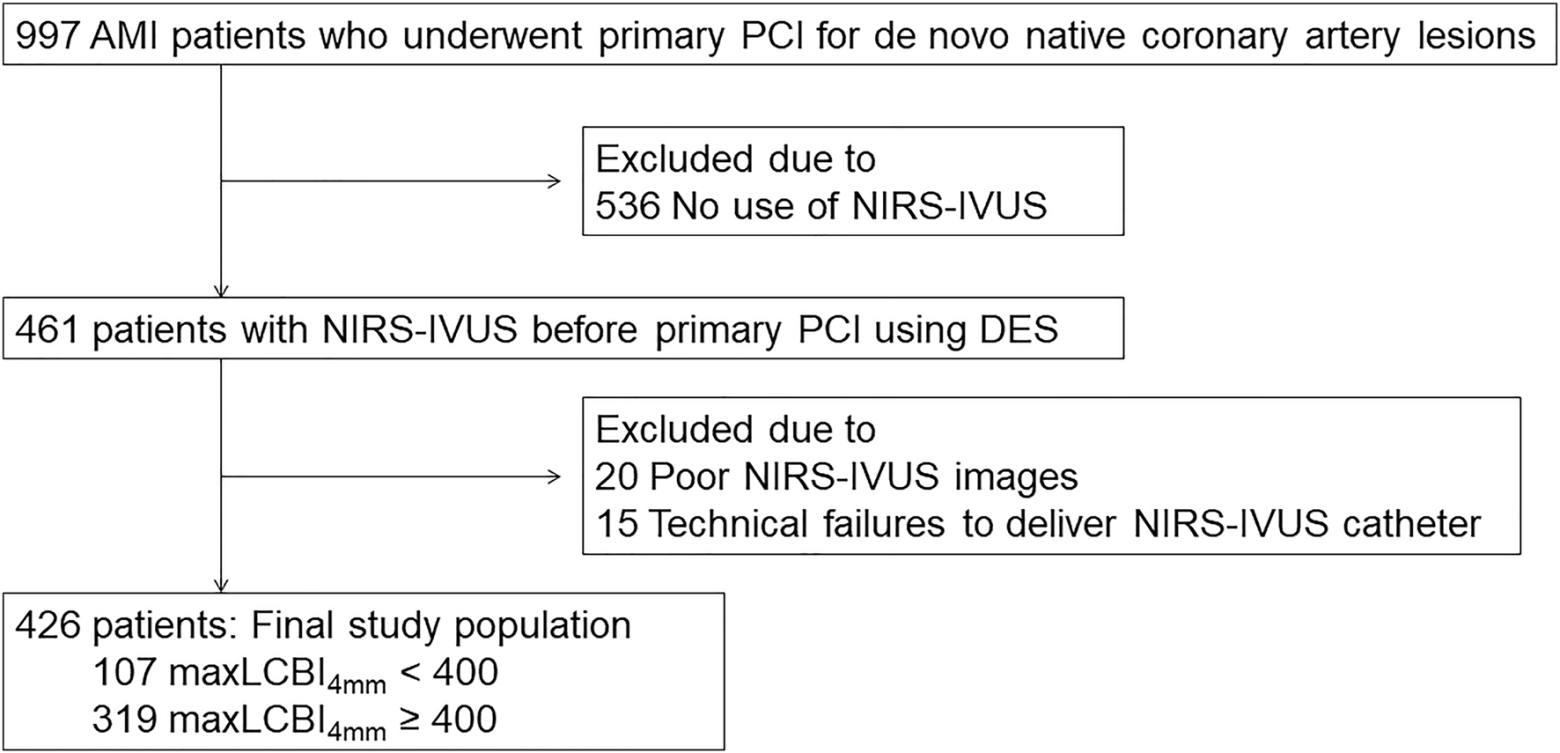
Patient selection. From 997 patients with AMI, 426 patients who underwent NIRS-IVUS before primary PCI were selected for analysis. AMI: acute myocardial infarction, DES: drug-eluting stent, IVUS: intravascular ultrasound, LCBI: lipid core burden index, NIRS: near-infrared spectroscopy, PCI: percutaneous coronary intervention.

This was a multicentre retrospective registry of NIRS-IVUS in AMI patients. Between May 2016 and March 2021, 997 AMI patients underwent PCI for de novo native coronary artery lesions. NIRS-IVUS before PCI using a second-generation drug-eluting stent was performed in 461 patients (Supplementary Table 1). Of these, 35 patients were excluded due to technical failure to deliver NIRS-IVUS catheter (n = 20) and poor NIRS-IVUS images (n = 15). Thus, 426 patients constituted the final study population (Fig. 1). AMI was defined as a type 1 MI according to the universal definition^5^. The infarct-related lesion was identified by the operator at the time of angiography on the basis of angiographic lesion morphology, such as total or subtotal occlusion, as well as electrocardiographic findings and echocardiographic results. This study was carried out in accordance with the Declaration of Helsinki. This study was approved by the institutional review board at Wakayama Medical University (3384). The requirement for written informed consent was waived by the institutional review board at Wakayama Medical University because of the retrospective design of the study. All methods were carried out in accordance with relevant guidelines and regulations.

### NIRS-IVUS imaging

NIRS-IVUS was performed before PCI at the operator's discretion using the TVC Imaging System (MC8 with a 40-MHz TVC Insight catheter [n = 146] or MC10 with a 50-MHz Dualpro catheter [n = 280], InfraReDx, Burlington, Massachusetts, USA). There were no pre-determined inclusion/exclusion criteria for the use of NIRS-IVUS. In case with TIMI (Thrombolysis in Myocardial Infarction) flow grade 0 or 1^6^, balloon angioplasty with a small balloon ≤ 2.0 mm in diameter (n = 319 [75%]) and/or aspiration thrombectomy with a 5.1-F aspiration catheter (n = 132 [31%]) was performed prior to the NIRS-IVUS imaging. The NIRS-IVUS catheter was advanced distally to the infarct-related lesion over a 0.014-inch conventional angioplasty guidewire. The NIRS-IVUS imaging core was retracted to the coronary ostia at a rate of 0.5 mm/s for the 40-MHz TVC Insight catheter or 2.0 mm/s for the 50-MHz Dualpro catheter using an automatic pullback device. Color-coded NIRS spectral data co-registered with IVUS images were acquired during pullback and stored digitally for offline analysis.

### NIRS-IVUS analysis

NIRS-IVUS analysis was performed using CAAS Intra Vascular (Pie Medical Imaging, Maastricht, the Netherlands) in an independent core laboratory (Wakayama Medical University, Wakayama, Japan), blinded to clinical information and angiography findings. In IVUS, plaque rupture (defined by an intra-plaque cavity that communicated with the lumen with an overlying residual fibrous cap fragment)^7^, attenuated plaque (defined by a signal reduction behind hypoechoic plaque without calcium), and convex calcium (defined by an intraluminal protrusion > 0.5 mm in thickness, with a bright echo, convex shape, irregular surface, and acoustic shadowing) were evaluated^3^. External elastic membrane (EEM), lumen, and plaque burden (defined as [EEM area—lumen area]/EEM area × 100) were measured on a cross-section at 1 mm longitudinal intervals^8^. Minimum lumen area (MLA) was determined in the infarct-related lesion. Reference site was set at a cross-section adjacent to the infarct-related lesion that had the largest lumen and plaque burden of < 50%^8^. Lesion length was defined as a distance between the proximal and distal reference sites^8^. Positive remodeling was defined by a remodeling index (calculated as EEM area at the MLA site/the proximal reference EEM area) > 1.05^9^.

NIRS data were automatically displayed on a chemogram demonstrating the distribution of lipid within the coronary artery in a longitudinal view. In the chemogram, lipid content was quantified as the LCBI, defined as the fraction of valid pixels indicating lipid at the probability > 0.6 within the scanned region multiplied by 1000. The LCBI_vessel_ was defined as the value of LCBI in the entire scanned region within the infarct-related vessel^2^. The maxLCBI_4mm_ was defined as the maximum value of the LCBI for any 4 mm region in the infarct-related lesion^2^.

### PCI

PCI was performed in a standard manner using a second-generation drug-eluting stent (Supplementary Table 2). During PCI, patients received intravenous heparin (a bolus of 100 IU/kg and additional doses aimed at achieving activated clotting time of 250–300 s). Thrombolysis was not performed for any patient. Glycoprotein IIb/IIIa inhibitors were not used because they were not approved in Japan. Dual antiplatelet therapy with aspirin and a thienopyridine (clopidogrel or prasugrel) was continued at least 6 months after PCI.

### Angiography

Angiograms before and immediately after PCI were analysed using QAngio XA ver 7.1 (Medis, Leiden, the Netherlands) in an independent core laboratory (Wakayama Medical University, Wakayama, Japan), blinded to clinical information and NIRS-IVUS findings. Quantitative coronary angiography included the reference lumen diameter, minimum lumen diameter, and percent diameter stenosis. Multivessel disease was defined as having angiographical stenoses with more than 50% diameter stenosis in two or three major epicardial coronary arteries. TIMI flow grade before PCI, no-reflow (defined as TIMI flow grade 0, 1 or 2) immediately after PCI and distal embolization (defined as a distal filling defect with an abrupt cut-off in the infarct-related artery) immediately after PCI were evaluated according to established definitions^6^. Balloon-to-artery ratio was defined as maximum balloon diameter divided by reference lumen diameter.

### Clinical outcomes

Clinical follow-up data of patients were collected from their medical records. The adjudication of major adverse cardiovascular events (MACE) was based on the discussions with three experienced cardiologists (Y.S, T.Ku, and S.F) who were blinded to the NIRS-IVUS results. MACE was defined as the composite of cardiac death^10^, non-fatal MI (including culprit plaque [CP] -related MI and non-culprit plaque [NCP] -related MI)^5^, clinically driven target lesion revascularization (TLR)^10^, clinically driven non-TLR^10^, or congestive heart failure (CHF) requiring hospitalization^11^. TLR was defined as a repeat revascularization for the lesions treated with baseline PCI. Non-TLR was defined as a revascularization for non-infarct-related lesions that were identified in baseline coronary angiography. Scheduled revascularization for non-infarct-related lesions was not considered as an adverse event. TLR/non-TLR was considered to be clinically-driven if revascularization was performed on a patient who had a positive result on a functional ischemia study and ischemic symptoms such as chest pain^10^.

### Statistical analysis

Statistical analysis was performed by using JMP 13.0 (SAS Institute, Cary, North Carolina, USA). Categorical variables were presented as frequency and percentages, with comparison with chi-square statistics or the Fisher exact test (if the expected cell value was < 5). Continuous variables were presented as median and interquartile range (IQR) and compared using the Mann–Whitney U test. The Kaplan–Meier method and log-rank test was used to compare the cumulative rate of MACE between the groups. Univariate Cox regression analysis was performed to evaluate for clinical, angiographic and NIRS-IVUS variables before PCI that were associated with MACE. The variables with *P*-values < 0.1 in the univariate analysis were then included in multivariable Cox regression analysis. Although both maxLCBI_4mm_ and LCBI_vessel_ met the criteria for inclusion in the multivariable Cox regression analysis, only maxLCBI_4mm_ was chosen for inclusion because maxLCBI_4mm_ more directly represents the tissue characteristics of infarct-related lesions. Results were reported as hazard ratios (HRs) with 95% confidence intervals (CIs). A *P*-value < 0.05 was considered statistically significant.

## Results

### Patient clinical characteristics

In NIRS before PCI, 107 (25%) patients had infarct-related lesions with maxLCBI_4mm_ < 400 (279 [IQR: 158–332]), and 319 (75%) patients had infarct-related lesions with maxLCBI_4mm_ ≥ 400 (710 [IQR: 556–827]). Patient clinical characteristics at baseline were not different between the maxLCBI_4mm_ < 400 group and the maxLCBI_4mm_ ≥ 400 group, except for age (68 years [IQR: 57–78 years] vs. 73 years [IQR: 64–80 years], *P* = 0.007) (Table 1 and Supplementary Table 3).

**Table 1:** Patient clinical characteristics.

	MaxLCBI_4mm_ < 400 (n = 107)	MaxLCBI_4mm_ ≥ 400 (n = 319)	*P*-value
Age, y	68 (57–78)	73 (64–80)	0.007
Male sex	83 (78)	243 (76)	0.768
Hypertension	81 (76)	224 (70)	0.277
Diabetes mellitus	38 (36)	119 (37)	0.740
Dyslipidaemia	39 (36)	116 (36)	0.987
Current smoking	32 (30)	105 (33)	0.564
Prior MI	6 (6)	21 (7)	0.720
Clinical presentation			
STEMI	79 (74)	232 (73)	0.824
NSTEMI	28 (26)	87 (27)	
Clinical findings			
Killip class 4	1 (1)	14 (4)	0.093
Peak CK-MB, IU/L	135 (33–277)	163 (52–337)	0.094
LVEF, %	48 (42–53)	45 (40–53)	0.212
Medications at discharge			
Aspirin	105 (98)	308 (97)	0.412
Thienopyridine	107 (100)	315 (99)	0.245
ACEI or ARB	96 (90)	280 (88)	0.589
β-blocker	78 (73)	237 (74)	0.776
Statin	103 (96)	303 (95)	0.589
Insulin	6 (6)	13 (4)	0.515
Medications at follow-up			
Aspirin	96 (90)	287 (90)	0.941
Thienopyridine	17 (16)	30 (9)	0.074
ACEI or ARB	97 (91)	266 (83)	0.067
β-blocker	93 (87)	280 (88)	0.816
Statin	102 (95)	294 (92)	0.268
Insulin	7 (7)	13 (4)	0.297

Values are presented as median (interquartile range) or number (%).

*ACEI* angiotensin converting enzyme inhibitor, *ARB* angiotensin II receptor blocker, *CK-MB* creatine kinase myocardial band, *LVEF* left ventricular ejection fraction measured by the modified Simpson’s method using transthoracic echocardiography at discharge, *LCBI* lipid core burden index, *NSTEMI* non-ST-segment elevation myocardial infarction, *STEMI* ST-segment elevation myocardial infarction.

### Angiographic findings and procedural characteristics

Angiographic findings and procedural characteristics are shown in Table 2. The distribution of infarct-related coronary artery was not different between the two groups. Reference lumen diameter, minimum lumen diameter, and percent diameter stenosis before PCI were similar between the two groups. The frequency of TIMI flow grade 0 or 1 before PCI (62% vs. 75%, *P* = 0.007) and multivessel disease (39% vs. 51%, *P* = 0.029) was significantly lower in the maxLCBI_4mm_ < 400 group compared with the maxLCBI_4mm_ ≥ 400 group.

**Table 2:** Angiographic findings and procedural characteristics.

	MaxLCBI_4mm_ < 400 (n = 107)	MaxLCBI_4mm_ ≥ 400 (n = 319)	*P*-value
Angiography before PCI			
LMCA/LAD/LCX/RCA	0/53/14/40	0/163/49/107	0.718
Reference lumen diameter, mm	3.20 (2.85–3.63)	3.15 (2.79–3.50)	0.219
Minimal lumen diameter, mm	0 (0.09–0.30)	0 (0–0.20)	0.116
Percent diameter stenosis, %	99 (92–100)	100 (94–100)	0.062
TIMI flow grade 0/1/2/3, %	55 (51)/11 (10)/23 (21)/18 (17)	181 (57)/59 (18)/54 (17)/25 (8)	0.012
Multivessel disease	42 (39)	164 (51)	0.029
Procedures			
Stent diameter, mm	3.50 (3.00–3.50)	3.25 (2.75–3.50)	0.100
Stent length, mm	18 (16–24)	24 (18–33)	< 0.001
Max. balloon diameter, mm	3.50 (3.00–3.50)	3.25 (2.75–3.50)	0.076
Balloon-to-artery ratio	1.00 (0.96–1.07)	1.00 (0.94–1.06)	0.510
Angiography immediately after PCI			
Reference lumen diameter, mm	3.30 (3.00–3.72)	3.25 (3.00–3.50)	0.085
Minimal lumen diameter, mm	3.13 (2.78–3.45)	3.05 (2.75–3.40)	0.149
Percent diameter stenosis, %	5 (3–7)	4 (3–8)	0.723
No-reflow	5 (5)	35 (11)	0.039
Distal embolization	4 (4)	16 (5)	0.590

Values are presented as median (interquartile range) or number (%).

*LAD* left anterior descending artery, *LCX* left circumflex artery, *LMCA* left main coronary artery, *PCI* percutaneous coronary intervention, *RCA* right coronary artery, *TIMI* thrombolysis in myocardial infarction trial, other abbreviations as in Table 1.

Stent diameter was not different between the two groups. Stent length was significantly shorter in the maxLCBI_4mm_ < 400 group compared with the maxLCBI_4mm_ ≥ 400 group (18 mm [IQR: 16–24 mm] vs. 24 mm [IQR: 18–33 mm], *P* < 0.001). Maximum balloon diameter and balloon-to-artery ratio were similar between the two groups. Minimum lumen diameter and percent diameter stenosis immediately after PCI were not different between the two groups. The frequency of no-reflow was significantly lower in the maxLCBI_4mm_ < 400 group compared with the maxLCBI_4mm_ ≥ 400 group (5% vs. 11%, *P* = 0.039). The frequency of distal embolization was similar between the two groups.

### IVUS findings

IVUS findings before PCI are shown in Table 3. The frequency of plaque rupture (4% vs. 59%, *P* < 0.001) and attenuated plaque (23% vs. 77%, *P* < 0.001) was significantly lower in the maxLCBI_4mm_ < 400 group compared with the maxLCBI_4mm_ ≥ 400 group. The frequency of convex calcium was significantly higher in the maxLCBI_4mm_ < 400 group compared with the maxLCBI_4mm_ ≥ 400 group (14% vs. 6%, *P* = 0.008). Lesion length was significantly shorter in the maxLCBI_4mm_ < 400 group compared with the maxLCBI_4mm_ ≥ 400 group (17 mm [IQR: 14–22 mm] vs. 22 mm [IQR: 16–30 mm], *P* < 0.001). Reference EEM area was similar between the two groups. MLA was significantly larger in the maxLCBI_4mm_ < 400 group compared with the maxLCBI_4mm_ ≥ 400 group (2.5 mm^2^ [IQR: 2.1–3.2mm^2^] vs. 2.2 mm^2^ [IQR: 1.9–2.6mm^2^], *P* < 0.001). At the MLA site, EEM area (15.4 [IQR: 11.9–19.0] vs. 16.5 [IQR: 13.2–19.7], *P* = 0.039) and plaque burden (84% [IQR: 79–86%] vs. 87% [IQR: 83–89%], *P* < 0.001) were significantly smaller in the maxLCBI_4mm_ < 400 group compared with the maxLCBI_4mm_ ≥ 400 group. The frequency of positive remodeling (23% vs. 71%, *P* < 0.001) was significantly lower in the maxLCBI_4mm_ < 400 group compared with the maxLCBI_4mm_ ≥ 400 group.

**Table 3:** IVUS findings before PCI.

	MaxLCBI_4mm_ < 400 (n = 107)	MaxLCBI_4mm_ ≥ 400 (n = 319)	*P*-value
Plaque rupture, %	4 (4)	188 (59)	< 0.001
Attenuated plaque, %	25 (23)	245 (77)	< 0.001
Convex calcium, %	15 (14)	19 (6)	0.008
Lesion length, mm	17 (14–22)	22 (16–30)	< 0.001
Reference EEM area, mm^2^	15.8 (12.8–18.3)	14.4 (11.8–18.0)	0.072
MLA, mm^2^	2.5 (2.1–3.2)	2.2 (1.9–2.6)	< 0.001
EEM area at MLA site, mm^2^	15.4 (11.9–19.0)	16.5 (13.2–19.7)	0.039
Plaque burden at MLA site, %	84 (79–86)	87 (83–89)	< 0.001
Positive remodelling, %	25 (23)	228 (71)	< 0.001

Values are presented as median (interquartile range) or number (%).

*EEM* external elastic membrane, *MLA* minimum lumen area, other abbreviations as in Table 1.

### NIRS findings

The analyzed length of NIRS in the maxLCBI_4mm_ < 400 group and the maxLCBI_4mm_ ≥ 400 group was 65 mm [IQR: 55–75 mm] and 66 mm [IQR: 57–79 mm], respectively (*P* = 0.348). The value of LCBI_vessel_ was significantly different between the maxLCBI_4mm_ < 400 group and the maxLCBI_4mm_ ≥ 400 group (78 [IQR: 41–109] and 188 [IQR: 132–245], *P* < 0.001).

### Clinical outcomes

**Figure 2:**
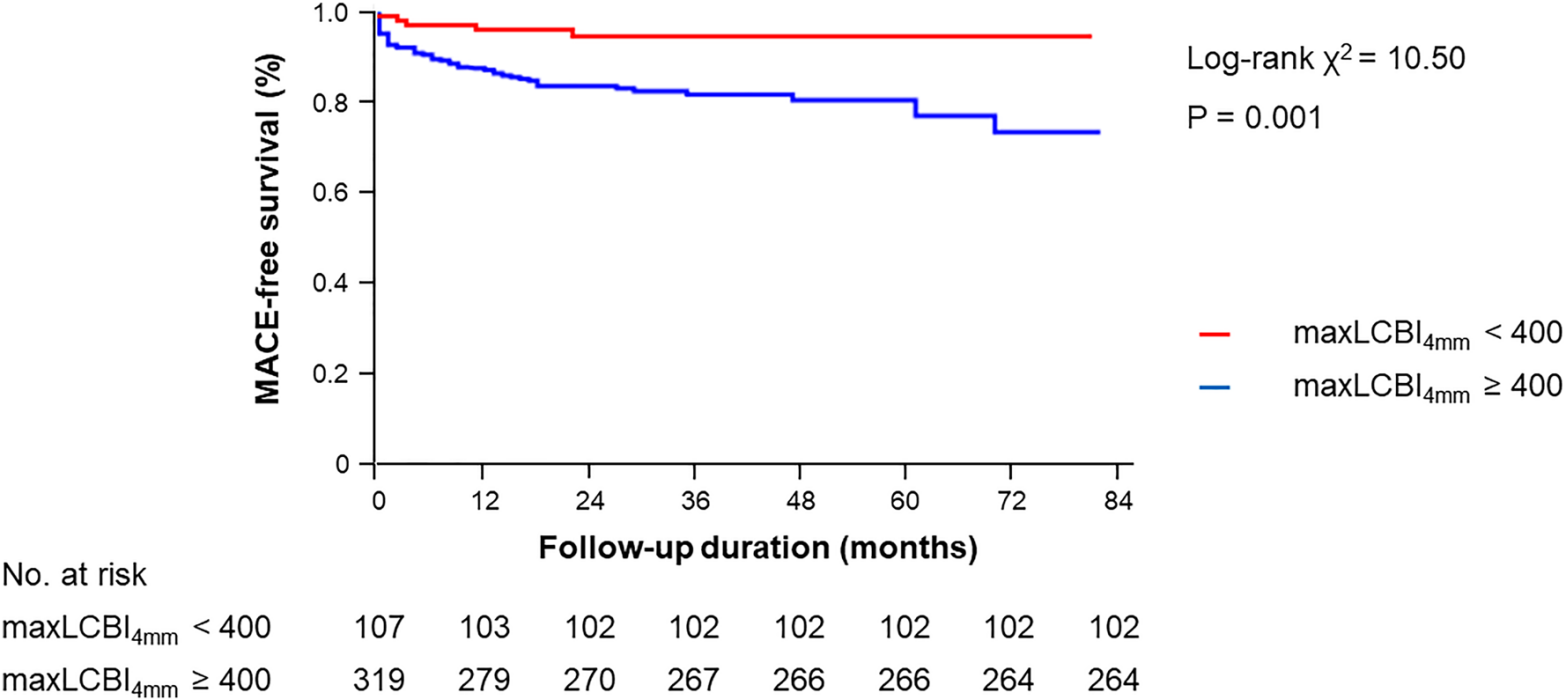
Kaplan–Meier curves of MACE-free survival. MACE-free survival was significantly better in the maxLCBI_4mm_ < 400 group (red line) compared with the maxLCBI_4mm_ ≥ 400 group (blue line) (*P* = 0.001). MACE: major adverse cardiovascular events.

Clinical outcomes are shown in Table 4. Follow-up duration in the maxLCBI_4mm_ < 400 group and the maxLCBI_4mm_ ≥ 400 group was 35 months [IQR: 18–52 months] and 30 months [IQR: 19–46 months], respectively (*P* = 0.222). The frequency of MACE was significantly lower in the maxLCBI_4mm_ < 400 group compared with the maxLCBI_4mm_ ≥ 400 group (4.7% vs. 17.2%, *P* = 0.001) (Fig. 2). The frequency of cardiac death (0% vs. 5.0%, *P* = 0.018) and non-fatal MI (0% vs. 4.4%, *P* = 0.028) was significantly lower in the maxLCBI_4mm_ < 400 group compared with the maxLCBI_4mm_ ≥ 400 group. The frequency of CP-related MI was not different between the two groups (0% vs. 1.3%, *P* = 0.245). The frequency of NCP-related MI was numerically lower in the maxLCBI_4mm_ < 400 group compared with the maxLCBI_4mm_ ≥ 400 group, but the differences did not reach statistical significance. (0% vs. 3.1%, *P* = 0.064). The frequency of TLR, non-TLR, and CHF requiring hospitalization was not different between the two groups.

**Table 4:** MACE.

	MaxLCBI_4mm_ < 400 (n = 107)	MaxLCBI_4mm_ ≥ 400 (n = 319)	*P*-value
MACE, %	5 (4.7)	55 (17.2)	0.001
Cardiac death	0 (0)	16 (5.0)	0.018
Non-fatal MI, %	0 (0)	14 (4.4)	0.028
CP-related MI, %	0 (0)	4 (1.3)	0.245
NCP-related MI, %	0 (0)	10 (3.1)	0.064
TLR, %	1 (0.9)	2 (0.6)	0.742
Non-TLR, %	1 (0.9)	8 (2.5)	0.327
CHF, %	3 (2.8)	15 (4.7)	0.398

Values are presented as number (%).

*CHF* congestive heart failure requiring hospitalization, *CP* culprit plaque, *MACE* major adverse cardiovascular event, *NCP* non-culprit plaque, *TLR* target lesion revascularization, other abbreviations as in Table 1.

Regarding the prediction of MACE, diabetes mellitus, prior MI, Killip class 4, peak creatine kinase myocardial band > 200 IU/L, left ventricular ejection fraction < 40%, multivessel disease, maxLCBI_4mm_ < 400, attenuated plaque, lesion length > 20 mm, and positive remodeling were variables with a *p*-value < 0.10 in univariate Cox regression analysis (Supplementary Table 5). The multivariable analysis including these variables revealed that Killip class 4 was an independent predictor of MACE (HR: 4.79, [CI: 1.88–12.25], *P* = 0.001) and the maxLCBI_4mm_ < 400 was an independent predictor of MACE-free survival (HR: 0.36 [CI: 0.13–0.98], *P* = 0.046) (Table 5).

**Table 5:** Multivariable Cox regression analysis for MACE.

	HR	95% CI	*P*-value
Diabetes mellitus	1.74	0.99–3.05	0.052
Prior MI	2.15	0.96–4.82	0.064
Killip class 4	4.79	1.88–12.25	0.001
Peak CK-MB ≥ 200 IU/L	1.64	0.93–2.91	0.090
LVEF < 40%	1.66	0.82–3.37	0.160
Multivessel disease	1.57	0.90–2.74	0.114
MaxLCBI_4mm_ < 400	0.36	0.13–0.98	0.046
Attenuated plaque	1.10	0.56–2.13	0.784
Lesion length > 20 mm	1.31	0.75–2.28	0.345
Positive remodelling	1.18	0.63–2.19	0.609

*CI* confidence interval, *HR* hazard ratio. Other abbreviations as in Table 1.

## Discussion

The major findings in the present NIRS-IVUS study were that the AMI patients with maxLCBI_4mm_ < 400 in infarct-related coronary lesions had a younger median age at onset, less frequent multivessel disease, less frequent TIMI flow grade 0 or 1 before PCI, less frequent no-reflow immediately after PCI, and better long-term clinical outcomes compared with those with maxLCBI_4mm_ ≥ 400. In addition, maxLCBI_4mm_ < 400 in infarct-related coronary lesions was an independent predictor of MACE-free survival.

### Infarct-related lesions with low LCBI

Most infarct-related lesions of AMI show high LCBI in NIRS. A previous NIRS study demonstrated that the infarct-related plaques had a median maxLCBI_4mm_ of 523 (IQR: 445–821) and a threshold of maxLCBI_4mm_ > 400 distinguished infarct-related plaques from non-infarct-related plaques with a sensitivity of 85% and specificity of 98%^2^. Infarct-related plaques with low maxLCBI_4mm_ are likely to have different pathologies compared with those with high maxLCBI_4mm_. An autopsy study demonstrated that pathological intimal thickening without lipid-core is the basis of plaque erosion rather than plaque rupture^1^. A case report showed that maxLCBI_4mm_ in plaque erosion was zero^12^. Our recent clinical study revealed that maxLCBI_4mm_ was significantly smaller in plaque erosion compared with plaque rupture and maxLCBI_4mm_ < 426 identified plaque erosion with sensitivity 92%, specificity 97%^3^. Therefore, the maxLCBI_4mm_ < 400 group in the present study may consist mostly of plaque erosion.

### Acute results of PCI

Acute results of PCI for a lipid-free or small lipid-containing plaques in AMI appears to be associated with better clinical outcomes than those for lipid-rich plaques. A NIRS-IVUS study showed that lower maxLCBI_4mm_ in the infarct-related lesions was associated with smaller infarction size and less frequent coronary microvascular obstruction assessed by magnetic resonance imaging after PCI^13^. In the present study, the maxLCBI_4mm_ < 400 group had a lower frequency of no-reflow phenomenon during PCI than the maxLCBI_4mm_ ≥ 400 group. The favourable acute results of PCI for a lipid-free or small lipid-containing plaques in AMI might have a positive effect on long-term outcomes.

### Long-term clinical outcomes

Pathohistological characteristics of infarct-related lesions are associated with long-term clinical outcomes in AMI patients treated with PCI. Previous OCT studies have demonstrated that AMI arising from a lipid-free or small lipid-containing plaque with intact fibrous cap (i.e. OCT-derived plaque erosion) had a lower frequency of MACE (including death, MI, revascularization, and CHF) during a follow-up period of > 1 year compared with AMI arising from lipid-rich plaque with disrupted fibrous-cap^14–16^. In line with these OCT studies, the present NIRS-IVUS study showed that lower maxLCBI_4mm_ in the infarct-related plaques was associated with better long-term clinical outcomes in AMI patients. One reason for this association is that maxLCBI_4mm_ in the infarct-related plaques may reflect the amount of remaining vascular lipid after PCI in three coronary arteries. In the present study, multivessel disease was less frequent in the maxLCBI_4mm_ < 400 group compared with the maxLCBI_4mm_ ≥ 400 group. The extent of coronary atherosclerosis might influence long-term outcomes. Based on our results, the patients with maxLCBI_4mm_ ≥ 400 in the infarct-related plaques might require stricter clinical follow-up and aggressive treatment of coronary risk factors to prevent future MACE. Recent clinical studies have shown that the intensive lipid-lowering therapy with proprotein convertase subtilisin kexin type 9 inhibitor (PCSK9i) increased fibrous cap thickness and decreased lipid core volume in coronary atheroma^17,18^. In patients with maxLCBI_4mm_ ≥ 400 in the infarct-related plaques, aggressive use of PCSK9i should be considered to stabilize atherosclerotic plaques and prevent future cardiovascular events.

### Limitations

There are several limitations that should be acknowledged. First, NIRS-IVUS was performed at the operator’s discretion, which might have led to selection bias. Second, there are limits to diagnosing plaque erosion using only LCBI in NIRS. Plaques with maxLCBI_4mm_ < 400 are not necessarily plaque erosion. Third, the present study did not use OCT. OCT allows accurate assessment of the morphological characteristics of the lesion responsible for AMI. The combined imaging of OCT and NIRS will be a promising technique for identifying plaque erosion. Fourth, balloon angioplasty and aspiration thrombectomy before imaging may have induced iatrogenic fibrous cap disruption and reduced the lipid composition in plaque rupture, although meticulous care was taken to avoid excessive mechanical trauma. Fifth, there were no data on NIRS-IVUS immediately after PCI. Therefore, the present study was unable to evaluate the clinical significance of changes in maxLCBI_4mm_ during PCI. Sixth, there were no data on NIRS-IVUS in the non-infarct-related vessel. The presence or absence of lipid-rich plaque in non-infarct-related vessels might affect the prognosis of the patient. Seventh, different types of stents were used in the present study. However, all were current generation drug-eluting stents, 80% of which were everolimus-eluting stent, and there were no statistical differences in stent type between the two groups. Finally, the patients receiving thienopyridine at follow-up tended to be more frequent in the maxLCBI_4mm_ < 400 group than in the maxLCBI_4mm_ ≥ 400 group, which may have influenced our results regarding clinical outcome.

## Conclusions

MaxLCBI_4mm_ < 400 measured by NIRS in the infract-related lesions before PCI was associated with better long-term clinical outcomes in AMI patients.

## Clinical perspectives

### Competency in medical knowledge

maxLCBI_4mm_ < 400 measured by NIRS in the infract-related lesions before PCI was associated with better long-term clinical outcomes in AMI patients.

### Translational outlook

NIRS might be useful in the risk stratification of AMI patients undergoing PPCI, because maxLCBI_4mm_ < 400 measured by NIRS in the infract-related lesions before PCI is associated with better long-term clinical outcomes in AMI patients.

## Supplementary Information

Supplementary Information.

## Data Availability

All data generated or analyzed during this study are included in this published article and its supplementary file.

## Abbreviations

AMI: Acute myocardial infarction
CHF: Congestive heart failure
EEM: External elastic membrane
IVUS: Intravascular ultrasound
LCBI: Lipid core burden index
maxLCBI_4mm_: Maximum lipid core burden index in 4 mm
MLA: Minimum lumen area
NIRS: Near-infrared spectroscopy
PCI: Percutaneous coronary intervention
TLR: Target lesion revascularization
