# Supplementary material for: Clinical outcomes of acute myocardial infarction arising from non-lipid-rich plaque determined by NIRS-IVUS
Source: Sci Rep. 2023 Jul 17;13:11544. doi: 10.1038/s41598-023-38578-9 (PMC10352267; doi:10.1038/s41598-023-38578-9)
Supplement: Supplementary file 1 — Supplementary Information. [file 41598_2023_38578_MOESM1_ESM.pdf]

Supplementary appendix

Clinical Outcomes of Acute Myocardial Infarction Arising from Non-lipid-rich Plaque determined by NIRS-IVUS

Kosei Terada, MD, PhD; Noriyuki Wakana, MD, PhD; Takashi Kubo, MD, PhD; Yasushi Ino, MD, PhD; Amir Kh. M. Khalifa, MD, PhD; Suwako Fujita, MD, PhD; Masahiro Takahata, MD, PhD; Yasutsugu Shiono, MD, PhD; Ryan D. Madder, MD; Takeyoshi Kameyama, MD, PhD

Table of contents	Page
1. Participating institutions and number of enrolled patients (Supplementary Table 1)	2
2. Stent type (Supplementary Table 2)	3
3. Serum LDL-C levels at baseline and at follow-up (Supplementary Table 3)	4
4. CP-related MIs (Supplementary Table 4)	5
5. Univariate Cox regression analysis for MACE (Supplementary Table 5)	6
6. ROC curve of $LCBI_{vessel}$ for predicting MACE (Supplementary Figure 1)	8

Supplementary Table 1. Participating institutions and number of enrolled patients

Participating institutions	Number of enrolled patients
1. Wakayama Medical University, Japan	377
2. Tohoku Medical and Pharmaceutical University, Japan	40
3. Kyoto Prefectural University of Medicine, Japan	44

Supplementary Table 2. Stent type

	$MaxLCBI_{4mm}$ < 400 (n=107)	$MaxLCBI_{4mm}$ ≥ 400 (n=319)	P-value
Everolimus-eluting stent			
Xience	70 (65)	195 (61)	0.428
Synergy	14 (13)	50 (16)	0.517
Promus	1 (1)	1 (0.3)	0.416
Sirolimus-eluting stent			
Ultimaster	13 (12)	41 (13)	0.850
Orsiro	3 (3)	13 (4)	0.549
Coroflex	1 (1)	0 (0)	0.096
Zotarolimus-eluting stent			
Resolute	4 (4)	14 (4)	0.772
Biolimus-eluting stent			
Nobori	1 (1)	5 (2)	0.631

Values are presented as number (%). LCBI: lipid core burden index.

Supplementary Table 3. Serum LDL-C levels at baseline and at follow-up

	$MaxLCBI_{4mm}$ < 400 (n=107)	$MaxLCBI_{4mm}$ ≥ 400 (n=319)	P-value
LDL-C at baseline, mg/dL	107 (85-129)	109 (91-136)	0.139
LDL-C at follow-up, mg/dL	69 (57-82)	67 (53-79)	0.321
Absolute change in LDL-C, mg/dL	40 (14-58)	43 (18-74)	0.194
Percentage change in LDL-C, %	37 (17-48)	39 (20-55)	0.247

Values are presented as median (interquartile range) or number (%). LDL-C: low-density lipoprotein cholesterol.

Supplementary Table 4. CP-related MIs

	Case 1	Case 2	Case 3	Case 4
Age, years	62	49	46	60
Male sex	Male	Male	Male	Male
Culprit vessel	LAD	LAD	LAD	RCA
Initial $maxLCBI_{4mm}$	809	923	873	520
Duration after initial PCI	29 months	4 months	6 months	6 months
Causes of MI	Stent thrombosis	Stent thrombosis	Stent thrombosis	Stent thrombosis

CP: culprit plaque, LAD: left anterior descending artery, LCBI: lipid core burden index, MI: myocardial infarction, PCI: percutaneous coronary intervention.

Supplementary Table 5. Univariate Cox regression analysis for MACE

	HR	95% CI	P-value
**Clinical variables**			
Age	1.02	0.99-1.04	0.161
Male sex	1.12	0.61-2.07	0.713
Hypertension	1.10	0.62-1.95	0.749
Diabetes mellitus	1.60	0.96-2.66	0.069
Dyslipidaemia	0.84	0.49-1.45	0.529
Current smoking	1.20	0.71-2.04	0.488
Prior MI	2.16	0.98-4.75	0.056
STEMI as clinical presentation	0.92	0.52-1.61	0.772
Killip class 4	7.69	4.41-13.41	<0.001
Peak CK-MB ≥200 IU/L	1.80	1.08-2.99	0.024
LVEF <40%	2.95	1.66-5.24	< 0.001
**Medications at discharge**			
Aspirin	0.93	0.13-6.76	0.947
Thienopyridine	0.46	0.06-3.30	0.438
ACEI or ARB	0.76	0.37-1.53	0.439
β-blocker	0.69	0.40-1.18	0.176
Statin	0.53	0.21-1.32	0.171
Insulin	1.48	0.54-4.10	0.448
**Angiographic variables**			
LAD as an infarct-related artery	0.91	0.54-1.53	0.718
TIMI flow grade 0 or 1 before PCI	1.19	0.67-2.11	0.557
Multivessel disease	2.08	1.22-3.54	0.007
**NIRS-IVUS variables**			
$MaxLCBI_{4mm}$ <400	0.25	0.10-0.62	0.003
$LCBI_{vessel}$ <138	0.45	0.25-0.81	0.005
Plaque rupture	1.25	0.75-2.07	0.391
Attenuated plaque	1.99	1.10-3.58	0.022
Convex calcium	1.08	0.43-2.69	0.873
Lesion length >20mm	1.66	1.00-2.76	0.050
Reference EEM area	2.33	0.47-10.48	0.286
MLA	2.59	0.33-17.18	0.344
EEM area at MLA site	2.32	0.46-10.43	0.290
Plaque burden at MLA site	1.09	0.22-6.31	0.918
Positive remodelling	1.62	0.94-2.79	0.084

ACEI: angiotensin converting enzyme inhibitor, ARB: angiotensin II receptor blocker, CK-MB: creatine

kinase myocardial band, CI: confidence interval, EEM: external elastic membrane, HR: hazard ratio, IVUS: intravascular ultrasound, LAD: left anterior descending artery, LCBI: lipid core burden index, MI: myocardial infarction, MACE: major adverse cardiovascular event, MLA: minimum lumen area, NIRS: near-infrared spectroscopy, TIMI: thrombolysis in myocardial infarction.

Supplementary Figure 1.

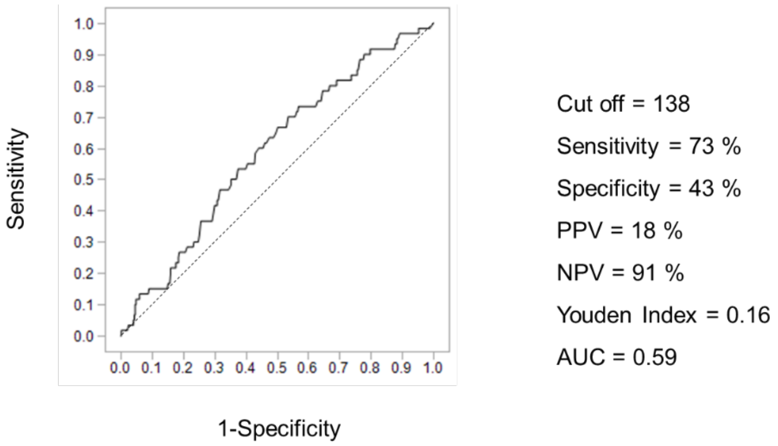

Title: ROC curve of $LCBI_{vessel}$ for predicting MACE

Caption: The $LCBI_{vessel}$ of 138 was the best cut-off to predict MACE.

AUC: area under the curve, ROC: receiver-operating characteristic, $LCBI_{vessel}$: lipid core burden index in the infarct-related vessel, MACE: major adverse cardiac event, NPV: negative predictive value, and PPV: positive predictive value.
